# Gram-negative bacterial infections in surgical intensive care unit patients following abdominal surgery: high mortality associated with *Stenotrophomonas maltophilia* infection

**DOI:** 10.1186/s13756-024-01411-7

**Published:** 2024-06-18

**Authors:** Ting-Lung Lin, Po-Hsun Chang, Yueh-Wei Liu, Wei-Hung Lai, Ying-Ju Chen, I-Ling Chen, Wei-Feng Li, Chih-Chi Wang, Ing-Kit Lee

**Affiliations:** 1 Department of Surgery, Kaohsiung Chang Gung Memorial Hospital, Kaohsiung, Taiwan; 2 Chang Gung University College of Medicine, Taoyuan, Taiwan; 3 Department of Pharmacy, Kaohsiung Chang Gung Memorial Hospital, Kaohsiung, Taiwan; 4 School of Pharmacy, Kaohsiung Medical University, Kaohsiung, Taiwan; 5 Division of Infectious Diseases, Department of Internal Medicine, Kaohsiung Chang Gung Memorial Hospital, Kaohsiung, Taiwan

**Keywords:** Surgical intensive care unit, Abdominal surgery, Gram-negative bacteria, *Stenotrophomonas maltophilia*, *Pseudomonas aeruginosa*, Mortality

## Abstract

**Background:**

*Stenotrophomonas maltophilia*, a multidrug-resistant gram-negative bacteria (GNB), is an emerging nosocomial pathogen. This study assessed the clinical outcomes of GNB infections in surgical intensive care unit (SICU) patients post-abdominal surgery, focusing on the differences between *S. maltophilia* and other GNBs, including *Pseudomonas aeruginosa*.

**Methods:**

A retrospective study was conducted on SICU patients at Kaohsiung Chang Gung Memorial Hospital from 2010 to 2020, who developed GNB infections following abdominal surgery.

**Results:**

Of 442 patients, 237 had *S. maltophilia* and 205 had non-*S. maltophilia* GNB infections (including 81 with *P. aeruginosa*). The overall mortality rate was 44.5%, and *S. maltophilia* infection emerged as a significant contributor to the mortality rate in patients with GNB infections. *S. maltophilia* patients had longer mechanical ventilation and SICU stays, with a 30-day mortality rate of 35.4%, higher than the non-*S. maltophilia* GNB (22.9%) and *P. aeruginosa* (21%) groups. In-hospital mortality was also higher in the *S. maltophilia* group (53.2%) compared to the non-*S. maltophilia* GNB (34.6%) and *P. aeruginosa* groups (29.6%). Risk factors for acquiring *S. maltophilia* included a higher Sequential Organ Failure Assessment score and prior broad-spectrum antibiotics use. Older age, polymicrobial infections, and elevated bilirubin were associated with increased 30-day mortality in *S. maltophilia* patients.

**Conclusion:**

*S. maltophilia* infections in post-abdominal surgery patients are linked to higher mortality than non-*S. maltophilia* GNB and *P. aeruginosa* infections, emphasizing the need for early diagnosis and treatment to improve outcomes.

## Introduction

Infections are prevalent among patients in intensive care units (ICU) and significantly contribute to mortality. The study on infection prevalence in ICU found that 51% of patients were infected, with gram-negative bacteria (GNB) identified as a frequent cause [1, 2]. Previous studies have highlighted *Pseudomonas aeruginosa* as a common nosocomial pathogen in the ICU setting, which carries a high mortality rate in patients, particularly when effective antibiotic therapy is delayed [3, 4]. However, the emergence of *Stenotrophomonas maltophilia*, an opportunistic GNB pathogen prevalent on surfaces within hospital environments, has increasingly been identified as a major culprit in ICU infections [5, 6]. *S. maltophilia*, inherently resistant to several classes of antibiotics, such as cephalosporins and carbapenems, is widely recognized as a challenging organism to treat [7]. Consequently, patients with *S. maltophilia* infections often face poor outcomes, including prolonged ICU stays and elevated mortality rates [8, 9]. Surgical patients, as a vulnerable population, necessitate heightened vigilance due to their augmented susceptibility to bacterial infections [10]. While *S. maltophilia* infections have been reported in ICU and trauma patients [11, 12], there is limited information available regarding the abdominal surgical population in the surgical ICU (SICU) setting, particularly in evaluating the impact of *S. maltophilia* infection on clinical outcomes. The objective of our study was to investigate the clinical features and outcomes of GNB infection, especially discerning the differences between *S. maltophilia* and non-*S. maltophilia* GNB, including *P. aeruginosa* infection, in SICU patients who underwent abdominal surgery. This information is particularly crucial in an era of advanced, complex surgical procedures and growing concerns about antimicrobial-resistant pathogens in the SICU, where timely and effective treatment may mitigate otherwise preventable morbidity and mortality in critically ill SICU patients.

## Methods

### Ethics approval

This study received approval from the Institutional Review Board of Kaohsiung Chang Gung Memorial Hospital (202200091B0C601), Taiwan. Informed consent was not necessary as the data were analyzed anonymously.

### Study design, setting, and participants

A retrospective study was conducted to analyze all consecutive adult patients (aged 20 years or older) who underwent abdominal surgery and were admitted to the SICU for three days or longer at Kaohsiung Chang Gung Memorial Hospital, a 2,600-bed primary care and tertiary academic medical center in Taiwan, from January 2010 to December 2020.

The exclusion criteria included patients who did not undergo abdominal surgery, those with gram-positive bacterial infections, and those without GNB infections following abdominal surgery. The inclusion criteria encompassed patients who underwent abdominal surgery and subsequently developed GNB infections. For patients who experienced multiple episodes of GNB infection during the same admission, only the initial GNB episode or the first episode of polymicrobial infection involving GNB was considered for analysis in this study. These patients were categorized into two groups: those with *S. maltophilia* infections and those with non-*S. maltophilia* GNB, including *P. aeruginosa* infections.

### Definitions

The surgical site classification in this study comprised four distinct areas. The first category encompassed the digestive system, which included the esophagus, stomach, duodenum, small intestine, colon, appendix, and rectum. The second category encompassed surgeries on the hepatobiliary system, pancreas, and spleen. The third category involved genitourinary surgeries, including the kidneys. Finally, the fourth category was focused on surgeries involving the abdominal wall. Antipseudomonal penicillins referred to piperacillin-tazobactam, while antipseudomonal cephalosporins included ceftazidime, cefoperazone-sulbactam, and cefepime. Carbapenems encompassed ertapenem, imipenem, meropenem, and doripenem. Fluoroquinolone referred to levofloxacin and ciprofloxacin. GNB infection is defined by the identification of GNB in a specimen from the affected site, supported by laboratory, radiological, and clinical evidence indicative of infection, sepsis, or septic shock [13]. Polymicrobial infection was defined as the concurrent isolation of multiple microorganisms, including GNB, from a blood specimen, along with clinical signs of infection. The in-hospital mortality refered to all-cause mortality that occurs during the hospital admission.

### Antimicrobial susceptibility testing

Microbiology laboratories performed antimicrobial susceptibility tests on the isolates using disk diffusion or automated testing methods (BD Phoenix™), following the guidelines and breakpoints outlined by the Institute of Clinical Laboratory and Standards [14]. Non-susceptibility was ascertained based on results obtained from in vitro antimicrobial susceptibility testing, indicating resistance, or intermediate susceptibility. The antimicrobial agents tested for *S. maltophilia* included trimethoprim/sulfamethoxazole, levofloxacin, moxifloxacin, and tigecycline, following the recommendations of Institute of Clinical Laboratory and Standards [14].

### Data collection

For our analysis, we collected a variety of variables including demographic information, Charlson’s comorbidity index [15], Sequential Organ Failure Assessment (SOFA) Score [16], the American Society of Anesthesiologists (ASA) physical status classification [17], surgical wound classification [18], types of surgical sites, duration of the surgical procedure, and any repeat surgeries during the same ICU stay. We also recorded data on various interventions, including mechanical ventilation, total parenteral nutrition, hemodialysis, blood transfusions, and catheter placements. Medication details, such as those pertaining to chemotherapy, immunosuppressants, steroids, and antibiotics, were gathered. Additionally, relevant laboratory results, including levels of albumin, alanine aminotransferase, creatinine, hemoglobin, platelet count, and total bilirubin, were taken into consideration. Lastly, we recorded outcomes such as the duration of mechanical ventilation, length of SICU stay, and both 30-day and in-hospital mortality rates post-GNB infection for a comprehensive analysis.

### Statistical analysis

Univariate and multivariate analyses were performed to evaluate the risk factors associated with all-cause mortality in patients with GNB infections included in the study. For a comprehensive understanding of GNB infection characteristics, univariate analysis was carried out to examine clinical and laboratory features, as well as outcomes, differentiating between patients with *S. maltophilia* and those with non-*S. maltophilia* GNB infections, such as *P. aeruginosa*. Furthermore, a comparative analysis between *S. maltophilia* and non-*S. maltophilia* GNB infections was performed to elucidate the risk factors predisposing patients to *S. maltophilia* infection. Kaplan-Meier curves were generated to compare 30-day survival between (a) patients with *S. maltophilia* infection and those with non-*S. maltophilia* GNB infection, and (b) patients with *S. maltophilia* infection and those with *P. aeruginosa* infection. Lastly, we conducted a comparative analysis between survivors and non-survivors to identify the independent risk factors associated with 30-day mortality after *S. maltophilia* infection.

Continuous variables are presented as means ± standard deviations, or median (interquartile range) while categorical variables are expressed as numbers and percentages. The Student’s t-test was employed for continuous variables, and the chi-square or Fisher’s exact test was utilized for categorical variables, as appropriate. Multivariate analysis was conducted utilizing a logistic regression model and a stepwise procedure to identify independent risk factors associated with the acquisition of *S. maltophilia* infection and 30-day mortality subsequent to *S. maltophilia* infection. All significance tests were two-sided, with a significance level set at *P* < 0.05. Statistical analyses were conducted using SAS EG version 5.1.

**Table 1:** Patient characteristics

Variables	Total GNB (*N* = 442)	*Stenotrophomonas maltophilia* (*n* = 237)	Non-*S. maltophilia* GNB (*n* = 205)	*Pseudomonas aeruginosa* (*n* = 81)	*P* ^a^	*P* ^b^
Mean age (SD) (years)	68 (14.8)	68.3 (15.2)	67.5 (14.4)	69.8 (13.7)	0.589	0.425
Age ≧ 65 years old, n (%)	290 (65.6)	157 (66.2)	133 (64.9)	58 (71.6)	0.763	0.374
Male, n (%)	270 (61.1)	153 (64.6)	117 (57.1)	55 (67.9)	0.108	0.585
BMI, mean (SD) (kg/m2)	23.6 (5.2)	23.3 (5.0)	23.9 (5.4)	23.7 (4.9)	0.221	0.517
Charlson score ≧ 3, n (%)	166 (37.6)	84 (35.4)	82 (40.0)	38 (46.9)	0.324	0.067
SOFA score, median (IQR)	5 (3–9)	6 (4–10)	5 (2–7)	4 (2–8)	< 0.001	< 0.001
Patient source from ED, n (%)	98 (22.2)	59 (24.9)	39 (19.0)	19 (23.5)	0.139	0.795
Hospital stay before admission to SICU, mean (SD) (days)	10.0 (16.8)	10.4 (18.1)	9.6 (15.3)	11.0 (16.5)	0.633	0.794
Emergency (non-elective) surgery, n (%)	221 (50.0)	135 (57.0)	86 (42.0)	30 (37.0)	0.002	0.002
ASA ≧ 3, n (%)	378 (85.5)	214 (90.3)	164 (80.0)	66 (81.5)	0.002	0.035
Surgical wound classification ≧ 3, n (%)	263 (59.5)	162 (68.4)	101 (49.3)	46 (56.8)	< 0.001	0.059
Surgical site						
Digestive system, n (%)	330 (74.7)	174 (73.4)	156 (76.1)	65 (80.3)	0.518	0.220
Hepatobiliary system, n (%)	109 (24.7)	56 (23.6)	53 (25.9)	16 (19.8)	0.588	0.472
Abdominal wall, n (%)	82 (18.6)	48 (20.3)	34 (16.6)	15 (18.5)	0.323	0.735
Genitourinary (including kidney) system, n (%)	48 (10.9)	27 (11.4)	21 (10.2)	8 (9.9)	0.699	0.707
Duration of the aforementioned operation, mean (SD) (mins)	294.6 (201.2)	289.5 (192)	300.5 (211.6)	265.3 (127.8)	0.566	0.202
Repeat surgery within the same admission, n (%)	105 (23.8)	69 (29.1)	36 (17.6)	15 (18.5)	< 0.001	0.062
Polymicrobial infection, n (%)	186 (42.1)	126 (53.2)	60 (29.3)	35 (43.2)	< 0.001	0.122
Length of mechanical ventilation^c^, mean (SD) (days)	6.5 (4.3)	8.0 (4.4)	4.0 (2.8)	3.8 (2.4)	< 0.001	< 0.001
Received total parenteral nutrition^c^, n (%)	217 (49.1)	113 (47.7)	52 (25.4)	22 (27.2)	< 0.001	< 0.001
Hemodialysis^c^, n (%)	58 (13.1)	43 (18.1)	15 (7.3)	6 (7.4)	< 0.001	0.021
Received blood transfusion^c^, n (%)	368 (83.3)	212 (89.5)	156 (76.1)	62 (76.5)	< 0.001	0.004
Catheters^c^						
Central venous catheter, n (%)	337 (76.2)	187 (78.9)	150 (73.2)	61 (75.3)	0.158	0.500
Double lumen catheter, n (%)	32 (7.2)	27 (11.4)	5 (2.4)	2(2.5)	< 0.001	0.016
Urinary catheter, n (%)	386 (87.3)	209 (88.2)	177 (86.3)	69 (85.2)	0.561	0.4820
Surgical drainage catheter, n (%)	344 (77.8)	196 (82.7)	148 (72.2)	63 (77.8)	0.008	0.325
Antibiotics therapy^c^						
Antipseudomonal penicillins, n (%)	89 (20.1)	67 (28.3)	22 (10.7)	7 (8.6)	< 0.001	< 0.001
Antipseudomonal cephalosporins, n (%)	42 (9.5)	33 (13.9)	9 (4.4)	2(2.5)	< 0.001	0.005
Carbapenems, n (%)	210 (47.5)	157 (66.2)	53 (25.9)	26 (32.1)	< 0.001	< 0.001
Fluoroquinolone, n (%)	40 (9.0)	32 (13.5)	8 (3.9)	0	< 0.001	< 0.001
Trimethoprim/sulfamethoxazole, n (%)	2(0.5)	1(0.4)	1(0.5)	0	1.000	0.558
Received chemotherapy^c^, n (%)	19 (4.3)	13 (5.5)	6 (2.9)	1(1.2)	0.186	0.089
Received immunosuppressant^c^, n (%)	5 (1.1)	4 (1.7)	1(0.5)	0	0.234	0.307
Received steroid^c, d^, n (%)	40 (9.0)	30 (12.7)	10 (4.9)	3 (3.7)	0.005	0.023
Laboratory data^c^						
WBC > 10,000/uL, (n%)	213 (48.2)	116 (49.0)	97 (47.3)	35 (43.2)	0.733	0.372
Hemoglobin < 8 g/dL, n (%)	27 (6.1)	12 (5.1)	15 (7.3)	5 (6.5)	0.324	0.755
Platelet count < 150 × 1000/µL, n (%)	173 (39.1)	96 (40.5)	77 (37.6)	27 (33.3)	0.527	0.253
Albumin < 2.5 mg/dL, n (%)	128 (29.0)	76 (32.1)	52 (25.4)	22 (27.2)	0.121	0.409
ALT > 80 U/L, n (%)	52 (11.8)	28 (11.8)	24 (11.7)	6 (7.4)	0.972	0.268
Creatinine > 1.2 mg/dL, n (%)	162 (36.7)	84 (35.4)	78 (38.1)	33 (40.7)	0.571	0.393
Total bilirubine > 1.4 mg/dL, n (%)	158 (35.7)	99 (41.8)	59 (28.8)	18 (22.2)	0.005	0.002

ALT, Alanine transaminase; ASA, American Society of Anesthesiologists; BMI, body mass index; ED, emergency department; GNB, gram-negative bacteria; IQR, interquartile range; SD, standard deviation; SOFA, Sequential Organ Failure Assessment; SICU, surgical intensive care unit

^a^Comparison between *S. maltophilia* and non-*S. maltophilia* GNB.

^b^Comparision between *S. maltophilia* and *P. aeruginosa*

^c^14-day preceding the occurrence of GNB infection

^d^Received steroid dosage ≧ 10 mg/day prednisolone-equivalent and ≧ 7 days

## Results

**Fig. 1:**
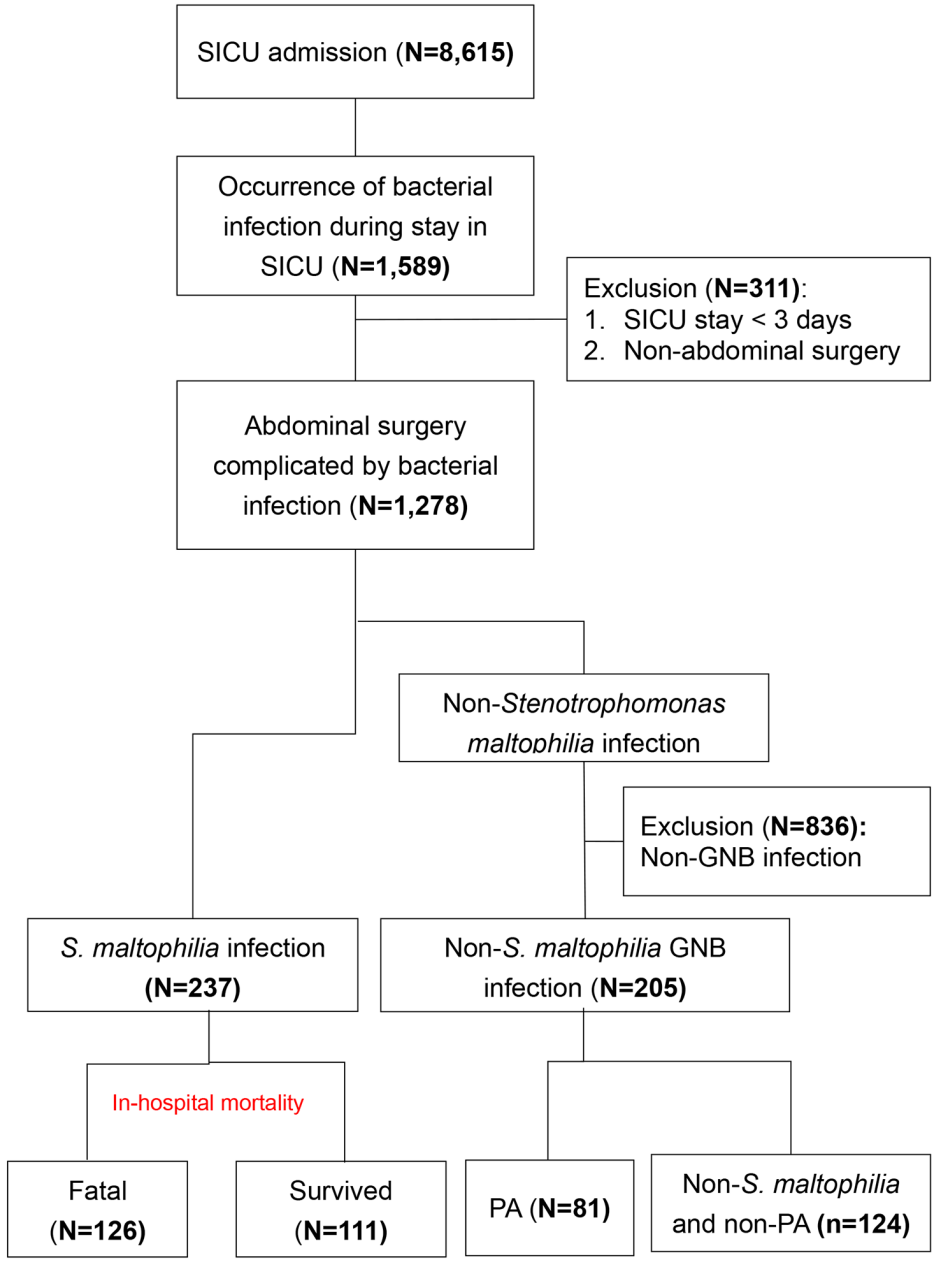
Patient flowchart. PA, *Pseudomonas aerugnosa*; SICU, surgical intensive care unit

Figure 1 presents the flowchart of the study. From a total of 1,278 patients who underwent abdominal surgery and subsequently developed bacterial infections, 442 were identified with GNB infections. Of these, 237 patients were diagnosed with *S. maltophilia* infection, while the remaining 205 had non-*S. maltophilia* GNB infections, which included 81 patients with *P. aeruginosa* infections. Table 1 details the characteristics of these 442 patients, categorizing them into those with *S. maltophilia* infections and those with non-*S. maltophilia* GNB infections, inclusive of *P. aeruginosa* infections.

### Risk factors associated with all-cause mortality among 442 patients with GNB infections (Table 2)

**Table 2:** Univariable and multivariable analysis of the risk factors associated with all-cause mortality in 442 patients with GNB infection

Variables	Fatal (*n* = 197)	Non-fatal (*n* = 245)	Univariable analysis	Multivariable analysis			
			*P*	Odds ratio	95% CI		*P*
Mean age (SD) (years)	69.5 (13.1)	66.7 (16.0)	0.043	1.017	1.003–1.031	0.021	
Male, n (%)	117 (59.4)	153 (62.5)	0.512				
BMI, mean (SD) (kg/m2)	23.3 (5.3)	23.8 (5.1)	0.306				
Charlson score, mean (SD)	2.4 (2.0)	1.9 (1.8)	0.014	1.118	1.007–1.242	0.037	
SOFA score, median (IQR)	6 (4–10)	5 (2–8)	< 0.001	1.104	1.041–1.171	0.001	
Patient source from ED, n (%)	46 (23.4)	52 (21.2)	0.593				
Hospital stay before admission to SICU, mean (SD) (days)	11.3 (20.6)	9.1 (13.0)	0.195				
Emergency (non-elective) surgery, n (%)	107 (54.3)	114 (46.5)	0.104				
ASA ≧ 3, n (%)	175 (88.8)	203 (82.9)	0.076				
Surgical wound classification ≧ 3, n (%)	129 (65.5)	134 (54.7)	0.022				
Surgical site							
Digestive system, n (%)	149 (75.6)	181 (73.9)	0.673				
Hepatobiliary system, n (%)	46 (23.4)	63 (25.7)	0.567				
Abdominal wall, n (%)	40 (20.3)	42 (17.1)	0.395				
Genitourinary (including kidney) system, n (%)	22 (11.2)	26 (10.6)	0.852				
Duration of the aforementioned operation, mean (SD) (mins)	274.8 (151.8)	310.6 (232.5)	0.052				
Repeat surgery within the same admission, n (%)	44 (22.3)	61 (24.9)	0.529				
Mechanical ventilation, mean (SD) (days)	7.3(4.4)	5.7(4.1)	< 0.001				
Received total parenteral nutrition, n (%)	84 (42.6)	81 (33.1)	0.039				
Hemodialysis, n (%)	35 (17.8)	23 (9.4)	0.010				
Received blood transfusion, n (%)	176 (89.3)	192 (78.4)	0.002	1.842	1.010–3.359	0.046	
Catheters							
Central venous catheter, n (%)	145 (73.6)	192 (78.4)	0.242				
Double lumen catheter, n (%)	21 (10.7)	11 (4.5)	0.013				
Urinary catheter, n (%)	170 (86.3)	216 (88.2)	0.557				
Surgical drainage catheter, n (%)	154 (78.2)	190 (77.6)	0.876				
*Stenotrophomona maltophilia* infecton, n (%)	126 (64.0)	111 (45.3)	< 0.001	1.573	1.022–2.420	0.039	
*Pseudomonas aeruginosa* infection, n (%)	24 (12.2)	57 (23.3)	0.003				

ASA, American Society of Anesthesiologists; BMI, body mass index; CI, confidential interval; ED, emergency department; GNB, gram-negative bacteria; IQR, interquartile range; SD, standard deviation; SOFA, Sequential Organ Failure Assessment; SICU, surgical intensive care unit

Among the 442 patients with GNB infections, 197 patients died, resulting in a 44.5% mortality rate among those with GNB infection. Univariable analysis revealed that mortality was significantly correlated with older age (69.5 vs. 66.7 years, *P* = 0.043), higher Charlson comorbidity index (2.4 vs. 1.9, *P* = 0.014), higher SOFA scores (6 vs. 5, *P* < 0.001), a higher proportion of patients with a surgical wound classification of 3 or above (65.5% vs. 54.7%, *P* = 0.022), prolonged mechanical ventilation (7.3 vs. 5.7, days *P* < 0.001), and increased rates of total parenteral nutrition (42.6% vs. 33.1%, *P* = 0.039), transfusion (89.3% vs. 78.4%, *P* = 0.002), hemodialysis (17.8% vs. 9.4%, *P* = 0.010), and double lumen catheter placement (10.7% vs. 4.5%, *P* = 0.013). Furthermore, *S. maltophilia* infections were significantly more prevalent among the deceased compared to the survivors (64% vs. 45.3%, *P* < 0.001). Conversely, *P. aeruginosa* infections were less frequent in deceased patients than in survivors (12.2% vs. 23.3%, *P* = 0.003).

Multivariable analysis indicated that older age (odds ratio [OR] 1.017, 95% confidence interval [CI] 1.003–1.031, *P* = 0.021), higher Charlson score (OR 1.118, 95% CI 1.007–1.242, *P* = 0.037), higher SOFA score (OR 1.104, 95% CI 1.041–1.171, *P* = 0.001), receipt of blood transfusion (OR 1.842, 95% CI 1.010–3.359, *P* = 0.046), and *S. maltophilia* infection (OR 1.573, 95% CI 1.022–2.420, *P* = 0.039) were independent risk factors for mortality in patients undergoing abdominal surgery with GNB infections. Table 2 presents the univariable and multivariable analyses for all-cause mortality in the 442 GNB-infected patients.

### Characteristics of patients with *S. maltophilia* infection (Table 1)

Among the 237 patients (mean age, 68.3 years) diagnosed with *S. maltophilia* infection, 59 patients (24.9%) were admitted through the emergency department, while emergency (non-elective) surgery was performed on 135 patients (57%). Regarding the surgical site, the digestive system was involved in 174 cases (73.4%), the hepatobiliary system in 56 cases (23.6%), the abdominal wall in 48 cases (20.3%), and the genitourinary system in 27 cases (11.4%). Of the 237 patients with *S. maltophilia* infection, 69 (29.1%) underwent repeat surgery during the same hospital admission. The predominant site of *S. maltophilia* infection was the respiratory tract, accounting for 61% of cases, followed by intrabdominal infections (24%), surgical wounds (11%), bloodstream (3%), and catheter-related infections (1%). Among the 237 *S. maltophilia* isolates, susceptibility rates were 95.3% for tigecycline, 94.4% for sulfamethoxazole/trimethoprim and moxifloxacin, and 88.4% for levofloxacin. Polymicrobial infection was identified in 126 patients (53.2%). Out of the 237 patients, 84 (35.4%) died within 30 days following the onset of *S. maltophilia* infection. In total, 126 out of the 237 patients died, yielding an in-hospital mortality rate of 53.2%.

### Characterisitics of patients with non-*S. maltophilia* GNB infection (Table 1)

A total of 205 patients (mean age 67.5 years) were identified with non-*S. maltophilia* GNB infection post-abdominal surgery. Among these, 39 patients (19.0%) were admitted through the emergency department, and 86 patients (42%) underwent emergency (non-elective) surgery. The predominant surgical site was the digestive system (76.1%), followed by the hepatobiliary system (25.9%), abdominal wall (16.6%), and genitourinary system (10.2%). The most commonly isolated non-*S. maltophilia* GNB were *P. aeruginosa* (28.8%), followed by *Escherichia coli* (14.9%), anaerobic GNB (13.5%), *Enterobacter cloacae* (8.5%), *Acinetobacter baumannii* (8.2%), and *Klebsiella pneumoniae* (7.1%) (Table 3). The predominant site for non-*S. maltophilia* GNB infections was the respiratory tract, comprising 35% of cases, followed by abdominal infections at 30%, surgical wounds at 23%, bloodstream infections at 11%, and catheter-related infections at 1%. Among these 205 patients, the 30-day mortality post-acquisition of non-*S. maltophilia* GNB infection was 22.9%, while the overall in-hospital mortality was 34.6%.

**Table 3:** The isolates of non-*Stenotrophomonas maltophilia* gram-negative bacteria

Pathogens	*N* (%)
*Pseudomonas aeruginosa*	81 (28.8)
*Escherichia coli*	42 (14.9)
Anaerobe gram-negative bacteria	38 (13.5)
*Enterobacter cloacae*	24 (8.5)
*Acinetobacter baumannii*	23 (8.2)
*Klebsiella pneumoniae*	20 (7.1)
Acinetobacter spp	13 (4.6)
Proteus spp	10 (3.5)
Non-fermenting gram-negative bacteria	7 (2.4)
Pseudomonas spp	6 (2.1)
Citrobacter spp	4 (1.4)
Enterobacter spp	4 (1.4)
Klebsiella spp	3 (1.1)
*Morganella morganii*	3 (1.1)
Providencia spp	2(0.7)
Serratia spp	1(0.4)

Out of 81 patients (mean age 69.8 years) with *P. aeruginosa* infections, 19 (23.5%) were admitted via the emergency department, and 30 (37.0%) underwent emergency (non-elective) surgery. The major surgical site was the digestive system, accounting for 80.3% of cases, followed by the hepatobiliary system (19.8%), abdominal wall (18.5%), and genitourinary system (9.9%). The most frequent sites of *P. aeruginosa* infection included the respiratory tract (47%), followed by surgical wounds (28%), the abdomen (20%), bloodstream (4%), and catheter-related infections (1%). The 30-day mortality rate post-acquisition of *P. aeruginosa* infection was 21.0%, with an overall in-hospital mortality rate of 29.6%.

### Comparative analysis of *S. maltophilia* and non-*S. maltophilia* GNB infections (Table 1)

Patients with *S. maltophilia* infections exhibited a significantly higher median SOFA score of 6 (interquartile range 4–10) compared to a median score of 5 (interquartile range 2–7) in the non-*S. maltophilia* GNB group (*P* < 0.001). A larger proportion of patients in the *S. maltophilia* group underwent emergency surgery compared to those in the non-*S. maltophilia* GNB group (57% vs. 42%, *P* = 0.002). Moreover, patients with *S. maltophilia* infections exhibited a significantly higher proportion of cases with ASA scores of ≧ 3 and surgical wound classifications of ≧ 3, as well as higher incidence of repeat surgery compared to those with non-*S. maltophilia* GNB infections (90.3% vs. 80.0% [*P* = 0.002], 68.4% vs. 49.3% [*P* < 0.001], and 29.1% vs. 17.6% [*P* < 0.001], respectively). The incidence of polymicrobial infections was significantly higher in patients with *S. maltophilia* infections compared to those with non-*S. maltophilia* GNB infections (53.2% vs. 29.3%, *P* < 0.001).

In terms of medical interventions, patients with *S. maltophilia* infections had a significantly higher likelihood of receiving total parenteral nutrition, hemodialysis, blood transfusions, double-lumen catheters, and surgical drainage catheters compared to those with non-*S. maltophilia* GNB infections, before the onset of their respective GNB infections (47.7% vs. 25.4% [*P* < 0.001], 18.1% vs. 7.3% [*P* < 0.001], 89.5% vs. 76.1% [*P* < 0.001], 11.4% vs. 2.4% [*P* < 0.001], and 82.7% vs. 72.2% [*P* = 0.008], respectively). Additionally, the *S. maltophilia* group showed significantly higher usage of antipseudomonal penicillins, antipseudomonal cephalosporins, carbapenems, fluoroquinolones, and steroids, and along with increased total bilirubin levels, compared to the non-*S. maltophilia* GNB group, prior to their GNB infections (28.3% vs. 10.7% [*P* < 0.001], 13.9% vs. 4.4% [*P* < 0.001], 66.2% vs. 25.9% [*P* < 0.001], 13.5% vs. 3.9% [*P* < 0.001], 12.7% vs. 4.9% [*P* < 0.005], and 41.8% vs. 28.8% [*P* = 0.005], respectively). Furthermore, the *S. maltophilia* group underwent significantly longer durations of mechanical ventilation compared to the non-*S. maltophilia* GNB group before the emergence of their respective GNB infections (8.0 ± 4.4 days vs. 4.0 ± 2.8 days, *P* < 0.001).

When comparing patients with *S. maltophilia* infections to those with *P. aeruginosa* infections, findings were largely parallel to those observed in the comparison with non-*S. maltophilia* GNB infections. However, there were exceptions in certain variables. These included surgical wound classification, the occurrence of repeat surgeries, hemodialysis, blood transfusions, the use of surgical drainage catheters, the presence of polymicrobial infections, and the use of antipseudomonal cephalosporins, where no statistical significance was observed. (Table 1).

### Comparative analysis of clinical outcomes among patients with *S. maltophilia*, non-*S. maltophilia* GNB, and *P. aeruginosa* infections (Table 4)

**Table 4:** Comparing the outcomes of patients with *Stenotrophomonas maltophilia* infection to those with infections caused by non-*S. maltophilia* gram-negative bacteria and *Pseudomonas aeruginosa*

	*S. maltophilia* (*n* = 237)	Non-*S.maltophilia* GNB (*n* = 205)	*Pseudomonas aeruginosa* (*n* = 81)	*P* ^a^	*P* ^b^
Length of mechanical ventilation after infection, mean (SD) (days)	14.4 (14.4)	9.7 (11.7)	9.4 (11.9)	< 0.001	< 0.001
Length of ICU stay after infection, mean (SD) (days)	19.7 (18.8)	15.9 (14.6)	15.3 (15.0)	0.018	0.011
Lenght of hospital stay, mean (SD) (days)	39.0 (35.5)	37.8 (33.2)	40.1 (42.1)	0.710	0.904
30-day mortality after infection, n (%)	84 (35.4)	47 (22.9)	17 (21.0)	0.004	0.016
In-hospital mortality, n (%)	126 (53.2)	71 (34.6)	24 (29.6)	< 0.001	< 0.001

GNB, gram-negative bacteria; ICU, intensive care unit; SD, standard deviation

^a^Comparison between *S. maltophilia* and non-*S. maltophilia* GNB.

^b^Comparision between *S. maltophilia* and *P. aeruginosa*

Patients with *S. maltophilia* infections exhibited significantly longer periods of mechanical ventilation and more extended stays in the SICU than those with non-*S. maltophilia* GNB infections (mean: 14.4 days vs. 9.7 days [*P* < 0.001], and mean: 19.7 days vs. 15.9 days [*P* = 0.018], respectively). Similarly, when compared with patients suffering from *P. aeruginosa* infections, the *S. maltophilia* group had longer mechanical ventilation (mean: 14.4 days vs. 9.4 days, *P* < 0.001) and SICU stays (mean: 19.7 days vs. 15.3 days, *P* = 0.011), respectively, following the onset of their respective GNB infections. The 30-day mortality rate post-GNB infections onset was 35.4% in the *S. maltophilia* group, which was significantly higher compared to 22.9% in the non-*S. maltophilia* GNB group (*P* = 0.004) and 21% in the *P. aeruginosa* group (*P* = 0.016). Moreover, the in-hospital mortality rate was significantly higher in the *S. maltophilia* group when compared to the non-*S. maltophilia* GNB (53.2% vs. 34.6%, *P* < 0.001) and *P. aeruginosa* (53.2% vs. 29.6%, *P* < 0.001) groups (Table 4).

**Fig. 2:**
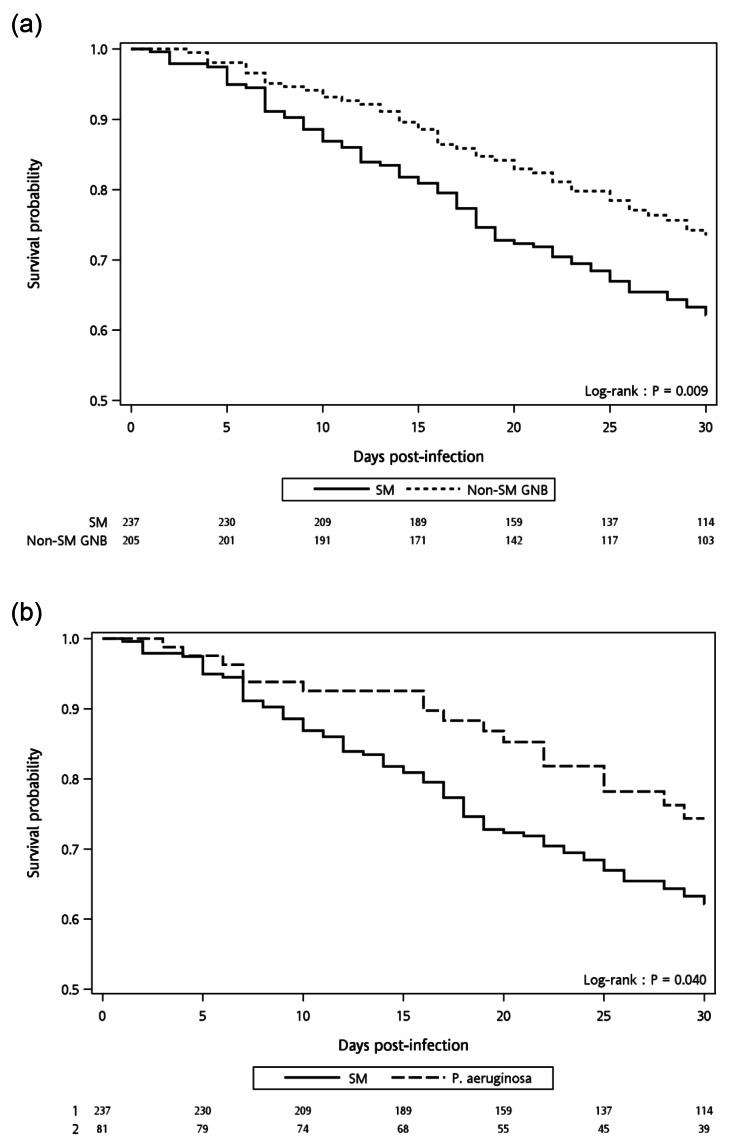
Kaplan-Meier curve comparing 30-day survival between (**a**) patients with *Stenotrophomonas maltophilia* infection and those with non-*S. maltophilia* gram-negative bacterial infection, and (**b**) patients with *S. maltophilia* infection and those with *Pseudomonas aeruginosa* infection

Kaplan-Meier survival analysis revealed a significantly lower 30-day survival rate in patients with *S. maltophilia* infection compared to those with non-*S. maltophilia* GNB infection (*P* = 0.009) (Fig. 2a), and those with *P. aeruginosa* infection (*P* = 0.04) (Fig. 2b).

### Independent risk factors for the acquisition of *S. maltophilia* infection following abdominal surgery

Multivariate analysis revealed that a high SOFA score (OR 1.087, 95% CI 1.019–1.160; *P* = 0.011) and the prior use of various antibiotics were independent risk factors for acquiring *S. maltophilia* infections in patients undergoing abdominal surgery. These antibiotics included antipseudomonal penicillin (OR 2.807, 95% CI 1.530–5.149; *P* = 0.001), antipseudomonal cephalosporin (OR 3.952, 95% CI 1.650–9.468; *P* = 0.002), carbapenem (OR 4.637, 95% CI 2.853–7.536; *P* < 0.001), and fluoroquinolone (OR 3.841, 95% CI 1.499–9.845; *P* = 0.005).

### Independent risk factors for 30-day mortality among patients with *S. maltophilia* infection (Table 5)

**Table 5:** Comparing survivors and non-survivors within 30 days following *Stenotrophomonas maltophilia* infection

	Non-survivors (*n* = 84)	Survivors (*n* = 153)	Univariate analysis	Multivariate analysis		
			*P*	Odds ratio	95% CI	*P*
Mean age (SD) (years)	71.7 (12.3)	66.5 (16.4)	0.006	1.033	1.011–1.057	0.004
Male, n (%)	56 (66.7)	97 (63.4)	0.615			
BMI, mean (SD) (kg/m2)	23.0 (5.1)	23.5 (5.0)	0.472			
Charlson score ≧ 3, n (%)	38 (45.2)	46 (30.1)	0.020			
Immunosuppressant, n (%)	3 (3.6)	1(0.7)	0.129			
Steroid, n (%)	16 (19.1)	14 (9.2)	0.028			
SOFA score, median (IQR)	7 (3–9.5)	6 (4–10)	0.801			
Patient source from ED, n (%)	22 (26.2)	37 (24.2)	0.732			
Emergency (non-elective) surgery, n (%)	48 (57.1)	87 (56.9)	0.967			
ASA ≧ 3, n (%)	75 (89.3)	139 (90.9)	0.697			
Surgical site						
Digestive system, n (%)	61 (72.6)	113 (73.9)	0.837			
Hepatobiliary system, n (%)	21 (25.0)	35 (22.9)	0.713			
Abdominal wall, n (%)	19 (22.6)	29 (19.0)	0.502			
Genitourinary (including kidney) system, n (%)	10 (11.9)	17 (11.1)	0.854			
Duration of the aforementioned operation, mean (SD) (mins)	267 (152.3)	301.9 (210.0)	0.144			
Repeat surgery within the same admission, n (%)	18 (21.4)	36 (23.5)	0.712			
Length of mechanical ventilation, mean (SD) (days)	23 (11.0)	27.3 (26.6)	0.084			
Received total parenteral nutrition, n (%)	65 (77.4)	114 (74.5)	0.623			
Hemodialysis, n (%)	30 (35.7)	42 (27.5)	0.186			
Inappropriate empirical antimicrobial therapy for *S. maltophilia*, n (%)	43 (51.2)	74 (48.4)	0.677			
Received tigecycline or quinolone within 48 h after occurrence *S. maltophilia* infection	58 (69.1)	114 (74.5)	0.367			
Received trimethoprim/sulfamethoxazole within 48 h after occurrence *S. maltophilia* infection	2 (2.4)	4 (2.6)	0.692			
Polymicrobial infection, n (%)	58 (69.1)	68 (44.4)	< 0.001	2.510	1.376–4.577	0.003
Site of *S. maltophilia* infection						
Respiratory tract	51 (60.7)	100 (65.4)	0.477			
Abdomen	21 (25.0)	40 (26.1)	0.847			
Surgical wound	11 (13.1)	16 (10.5)	0.541			
Blood	2 (2.4)	5 (3.3)	0.777			
Indwelling catheter	2 (2.4)	1(0.7)	0.287			
Laboratory data (one week within occurrence *S. maltophilia* infection)						
Mean WBC (SD), x 1000/uL	16.0 (9.4)	12.7 (6.2)	0.012			
Mean hemoglobin (SD), g/dL	9.2 (1.3)	9.8 (1.3)	0.002			
Mean platelet count (SD), x 1000/µL	120.8 (108.6)	221.6 (153.6)	< 0.001			
Mean albumin (SD), mg/dL	2.5 (0.5)	2.6 (0.5)	0.274			
Mean ALT (SD), U/L	48.6 (88.5)	54.5 (86.6)	0.622			
Mean creatinine (SD), mg/dL	2.3 (2.1)	1.8 (1.9)	0.077			
Mean total bilirubine (SD), mg/dL	5.8 (7.5)	3.1 (3.7)	0.003	1.105	1.039–1.176	0.002

ALT, Alanine transaminase; ASA, American Society of Anesthesiologists; BMI, body mass index; CI, confidential interval; ED, emergency department; IQR, interquartile range; SD, standard deviation; SOFA, Sequential Organ Failure Assessment

Upon comparing survivors and non-survivors within 30 days post *S. maltophilia* infection, it was observed that non-survivors were significantly older, had a higher proportion of Charlson scores ≥ 3 and steroid usage, exhibited a higher rate of polymicrobial infection, lower hemoglobin levels and platelet counts, as well as elevated total bilirubin levels compared to survivors (mean age: 71.7 years vs. 66.5 years [*P* = 0.006], 45.2% vs. 30.1% [*P* = 0.020], 69.1% vs. 44.4% [*P* < 0.001], mean: 9.2 g/dL vs. 9.8 g/dL [*P* = 0.002], mean: 120.8 × 1000/µL vs. 221.6 × 1000/µL [*P* < 0.001], and mean: 5.8 mg/dL vs. 3.1 mg/dL [*P* = 0.003], respectively) (Table 5).

Multivariate analysis revealed that older age (OR 1.033, 95% CI 1.011–1.057; *P* = 0.004), polymicrobial infection (OR 2.510, 95% CI 1.376–4.577; *P* = 0.003), and elevated total bilirubin levels (OR 1.105, 95% CI 1.039–1.176; *P* = 0.002) were identified as independent risk factors for 30-day mortality in patients who underwent abdominal surgery with *S. maltophilia* infection (Table 5).

## Discussion

Our study is the first to delve into the clinical characteristics of *S. maltophilia* infections, comparing them with other non-*S. maltophilia* GNB and *P. aeruginosa* infections, and assessing their impact on clinical outcomes in SICU patients post-abdominal surgery. Our findings illuminate several critical insights. Firstly, there is a striking 44.5% mortality rate among abdominal surgical patients with GNB infection, with *S. maltophilia* infections occurring significantly more frequently in fatal cases than in survivors. Secondly, patients with *S. maltophilia* infection exhibited higher SOFA scores and were more frequently administered broad-spectrum antibiotics prior to the onset of the *S. maltophilia* infection. Thirdly, a lower 30-day survival rate and higher in-hospital mortality were observed in patients with *S. maltophilia* infection compared to those with non-*S. maltophilia* GNB and *P. aeruginosa* infections. Fourthly, old age, polymicrobial infection, and elevated total bilirubin levels were identified as independent risk factors for mortality among patients with *S. maltophilia* infection. These findings underscore the graver outcomes for abdominal surgery patients who develop *S. maltophilia* infection compared to those with other GNB infections, even when compared to the most common nosocomial pathogen, *P. aeruginosa*. This emphasizes the paramount importance of early diagnosis and treatment of *S. maltophilia* infection, as well as stringent infection control to mitigate the spread of *S. maltophilia* infection in an ICU setting.

Infections pose a pervasive challenge in ICUs, correlating with substantial morbidity and mortality [19, 20]. Within ICU settings, mortality rates linked to GNB infections exhibit considerable variability, typically fluctuating between 20% and 50%. This variation is influenced by the specific pathogen involved, the patient demographic, and the healthcare environment [1, 21]. Our series identified a notably high mortality rate of 44.5% among SICU patients who underwent abdominal surgery and subsequently developed a GNB infection. Not surprisingly, a high Charlson comorbidity index and SOFA score were identified as independent risk factors for mortality in these GNB patients. Furthermore, *S. maltophilia* infection emerged as a significant contributor to the mortality rate in patients with GNB infections.

A nationwide retrospective study in France, conducted by Guerci et al., revealed that 0.27% of 102,316 patients, admitted across 25 mixed ICUs over a three-year period, encountered hospital-acquired *S. maltophilia* pneumonia [22]. Similarly, a prospective observational case-control study by Nseir et al. demonstrated that 2% of 1,885 patients in a 30-bed mixed ICU developed ICU-acquired *S. maltophilia* colonization and/or infection [8]. In contrast, our study identified *S. maltophilia* infection in 18.5% (237 of 1278 patients) of abdominal surgical patients complicated with bacterial infection in the SICU, with these infections representing 53.6% (237 of 442 patients) of SICU-acquired GNB infections post-abdominal surgery. The elevated incidence of *S. maltophilia* infection in our study may be attributed to our specific emphasis on the abdominal surgical patient population, whereas previous studies enrolled patients from mixed ICU settings, encompassing varied patient population characteristics. The pronounced incidence of *S. maltophilia* infection in abdominal surgery patients serves as a reminder for physicians to maintain heightened vigilance regarding *S. maltophilia* infections when managing abdominal surgical patients in the ICU. This is especially crucial considering the intrinsic resistance of *S. maltophilia*, which might delay the initiation of effective antibiotic treatment and potentially lead to avoidable mortality and morbidity.

Our study highlights a pronounced association between elevated SOFA scores and the incidence of *S. maltophilia* infection. This correlation can be attributed to the frequent use of broad-spectrum antibiotics in these patients prior to the onset of *S. maltophilia* infection. Previous research has illuminated the role of broad-spectrum antibiotics as a pivotal risk factor for acquiring *S. maltophilia* infection. A retrospective study orchestrated by Imoto et al. pinpointed the administration of antipseudomonal β-lactams as a predictor of *S. maltophilia* infection [23]. Similarly, Hanes et al. identified that prior exposure to cefepime, an antipseudomonal cephalosporin, emerged as a risk factor for *S. maltophilia* infection in trauma ICU patients [12]. Furthermore, associations have been identified between the use of antipseudomonal cephalosporins and carbapenems and an elevated risk of *S. maltophilia* bacteremia [24]. The inherent resistance of *S. maltophilia* to β-lactam antibiotics, such as cephalosporins and carbapenems, amplifies the risk of infection or colonization. However, the administration of broad-spectrum antibiotics is often indispensable in critically ill patients in the ICU, especially those contending with intricate intraabdominal infections. Our findings underscore the critical need for the rigorous implementation of antimicrobial stewardship programs. These should encompass empirical antibiotic use guided by local guidelines and epidemiology, optimal dosing strategies, regular reviews of antimicrobial therapy in alignment with clinical progress and microbiological findings, de-escalation as soon as feasible, and timely cessation of therapy where appropriate, all with the overarching aim to mitigate the ecological impact on the patient’s microbiome and curtail the emergence of antimicrobial resistance.

Remarkably, the 30-day mortality rate for *S. maltophilia* infection surpassed that of other non-*S. maltophilia* GNB infections, even exceeding the rate for *P. aeruginosa*, a notorious nosocomial pathogen. Previous research has underscored the elevated mortality rates associated with *P. aeruginosa* infections, particularly among immunocompromised and critically ill patients [3, 25, 26]. *P. aeruginosa* bacteremia, acknowledged as a perilous nosocomial infection, frequently precipitates multi-site infections and exhibits a mortality rate oscillating between 18% and 36.4% in cases of bacteremia [27–29]. Consistent with these findings, our study identified a 30-day mortality rate of 21% among abdominal surgery patients with *P. aeruginosa* infection. Conversely, the 30-day mortality rate for *S. maltophilia* infection was a staggering 35.4%, with an overarching in-hospital mortality of 53.2%. Interestingly, the rate of polymicrobial infections involving both *S. maltophilia* and *P. aeruginosa* showed no significant difference, potentially impacting mortality analysis. Patients with *S. maltophilia* infections exhibited higher SOFA scores, required longer mechanical ventilation, and underwent more prolonged antibiotic therapy than those with *P. aeruginosa*, suggesting more severe clinical conditions and potentially increased mortality risk. The exact cause of the higher mortality in *S. maltophilia* patients—whether directly due to the *S. maltophilia* infection or underlying severe illnesses—remains unclear [30]. However, our study emphasizes that beyond the known risks of *P. aeruginosa*, there is a critical need for surgeons and intensivists to be vigilant and proactive in detecting *S. maltophilia* infections to improve patient outcomes.

Tunger et al. conducted a retrospective study on 35 episodes of *S. maltophilia* bacteremia in a tertiary academic center, pinpointing older age and renal insufficiency as mortality risk factors [31]. This aligns with a systematic review by Paez et al. in 2008, which highlighted organ dysfunction as an independent mortality risk factor post *S. maltophilia* infection [32]. Our findings resonate with these studies, emphasizing older age as a mortality predictor in *S. maltophilia* infection. Furthermore, we identified polymicrobial infection and elevated total bilirubin levels as significant independent predictors of 30-day mortality post *S. maltophilia* infection in ICU abdominal surgery patients. Notably, various studies have documented elevated mortality rates, ranging from 36 to 63%, in patients with polymicrobial bacteremia. These rates are influenced by patient comorbidities, diverse infection sources, and distinct causative pathogens [33–35]. Previous literature has reported a high rate of polymicrobial infections in *S. maltophilia* bacteremia [32], consistent with our observation that 69% of non-survivors developed polymicrobial infections post *S. maltophilia* infection. While the administration of inappropriate antimicrobial therapy did not significantly differ between survivors and non-survivors, the emergence of polymicrobial infections suggests a more intricate disease trajectory. Surgical-induced mucosal disruption might facilitate bacterial breakthrough infections. Conversely, elevated total bilirubin levels may signify not only hepatic dysfunction but also potential mechanical biliary tract obstruction and the progression of sepsis [36]. A previous study underscored that an elevation in serum bilirubin levels within the initial 72 h of admission is correlated with an augmented mortality risk in patients experiencing severe sepsis and septic shock [37]. Our findings accentuate the imperative of enhanced clinical vigilance in the management of GNB infections, especially those attributed to *S. maltophilia*.

Our study presents several limitations. Firstly, its retrospective nature inherently imposes constraints due to missing data on indicators of inflammation such as C-reactive protein and procalcitonin. Secondly, the inclusion of patients from a single institution may limit the generalizability of our findings to broader contexts. Thirdly, the absence of data on physicians’ clinical judgment concerning antimicrobial therapy might have restricted the depth of our analysis. Fourth, differences in the site of infection could potentially influence the outcomes of patients with *S. maltophilia* infection.

## Conclusion

In summary, GNB infection, particularly involving *S. maltophilia*, results in a high mortality rate among SICU patients who have undergone abdominal surgery. Our findings provide clinicians with a comprehensive understanding of the clinical features and risks of GNB infections, urging healthcare professionals to promptly identify high-risk patients. Given the considerable mortality rate attributed to *S. maltophilia* infection, initiating early, targeted treatment strategies are vital to enhancing patient outcomes.

## Data Availability

No datasets were generated or analysed during the current study.
